# The single and mixed impacts of cadmium, cobalt, lead, and PAHs on systemic immunity inflammation index in male and female

**DOI:** 10.3389/fpubh.2024.1356459

**Published:** 2024-02-15

**Authors:** Junfeng Nie, Zhumin Hu, Cuiyao Xian, Minxing He, Dengqiu Lu, Weipeng Zhang

**Affiliations:** Panyu Central Hospital, Guangzhou, Guangdong, China

**Keywords:** NHANES, cadmium, cobalt, lead, PAHs, systemic immunity inflammation index

## Abstract

**Background:**

Studies on the association between mixed exposure to common pollutants such as cadmium (Cd), cobalt (Co), lead (Pb), and polycyclic aromatic hydrocarbons (PAHs) with Systemic Immune Inflammatory Index (SII), a novel hemocyte-based inflammatory marker, have not been reported. This study explored the relationship between co-exposure to Cd, Co, Pb, PAHs, and SII.

**Methods:**

In this study, we used data from the National Health and Nutrition Examination Survey and enrolled adults with complete information on Cd, Co, Pb, PAHs, and SII. The linear regression was used to analyze the association of single pollutants with SII. Furthermore, a Bayesian Kernel Machine Regression analysis and a generalized weighted quantile sum regression analysis were used to analyze the association between mixed exposure to Cd, Co, Pb, and six PAHs and SII. We also separated males and females and analyzed the different effects of pollutants on SII, respectively.

**Results:**

5,176 participants were included in the study. After adjusting for age, gender, race, education, smoking, drinking, physical activity, and sedentary, Cd, Co, 1-OHN, 2-OHN and 2-OHF were positive with SII in the total population. Compared with the 50th percentile, the joint effect of pollutants on SII was positive. In the total population, males, and females, the top contaminant with the highest effect weights on SII were Co, Cd, and 1-OHN, respectively. The result of interaction analysis showed that the low concentrations of Cd had an elevation effect on SII in males.

**Conclusion:**

This study found a positive association of mixed exposure to Cd, Co, Pb, and six PAHs with SII, which occurred mainly in females.

## 1 Introduction

The systemic Immunity Inflammation Index (SII) proposed by Hu et al. for assessing the poor outcome of patients with hepatocellular carcinoma (1) is a novel inflammatory marker and can be used to evaluate systemic and local immune responses. The level of SII can be obtained by calculating the platelet count multiplied by the neutrophil count/lymphocyte count after the blood parameters have been tested, which is extremely convenient and cost-saving. The non-invasive SII reflects the changing *in vivo* levels and interactions of the three inflammatory cells and is more sensitive to the onset of an immune response than a single or two inflammatory cell indicator (2). As researchers have gradually recognized the importance of SII, an increasing number of reports have shown that SII is associated with many human health problems, such as hypertension (3), diabetes (4), and hyperlipidemia (5). The associations between SII and heart disease (6), kidney disease (7), and respiratory disease (8) have also been studied. As a relatively new marker of inflammation, much of the current research has focused on the relationship between SII and health problems, and research on the factors influencing SII is currently very limited. In the living environment, numerous factors such as harmful chemicals and metals can activate the immune system, leading to an inflammatory response in the body. The study by Wang et al. has shown that the toxicity of chemicals was associated with multi-organ interactions like the immune system, leading to inflammation (9). A cross-sectional study concluded that population exposure to PAH might affect the immunity produced by hepatitis vaccines (10). Sun et al. reported that exposure to heavy metal mixtures was positively correlated with immune responses, and there were synergistic and antagonistic effects of intermetallic interactions on immune responses (11). A study based on experiments with mice showed that interactions between heavy metals affected autoimmune diseases (12).

PAHs, a common family of chemicals, exist in coal, crude oil, and gasoline naturally and are produced by burning coal, oil, natural gas, wood, garbage, and tobacco (13). PAHs can also be made by the high temperatures in cooking and combined with or form small pellets in the environment (14). The human body could be exposed to PAHs through a variety of routes, such as inhaling contaminated air, consuming grilled or burned meat or food, and consuming food with airborne deposition of PAH particles, and some PAHs can pass through the skin and enter the body (15). When PAHs enter the body, the body will break down PAHs into metabolites that are subsequently excreted in urine or feces (16). The effects of exposure to PAHs on human health are multiorgan and multitissue. More than 100 categories of PAHs have been identified, and the United States Environmental Protection Agency lists the 16 most toxic PAHs as priority pollutants, which are carcinogens, mutagens, and teratogens, posing a serious threat to health (17). Some scientists have found that a variety of PAHs could affect blood parameters and the human immune system. A cohort study suggested that long-term exposure to PAH influenced the risk of cardiovascular disease through alterations in platelet counts (18). Parvez et al. have shown that PAH exposure could inhibit T cell division and impact the secretion of immune substances such as IL-2, IL-10, and IL-17A (19). PAH exposure may lead to the excessive secretion of pro-inflammatory mediators by macrophages, distributed to various organs like the circulatory system, inducing systemic inflammatory responses and further exacerbating localized pneumonia (20).

Heavy metals are also a group of widely distributed contaminants that are attached to various media, such as soil, drinking water, and suspended particulate matter in the air. Due to the prevalence of heavy metals in the soil and the widespread use of heavy metals in industry, agriculture, and medicine, people can be exposed to harmful heavy metals in a multitude of ways (21). Cadmium (Cd), cobalt (Co), and lead (Pb) are extensively accumulated in the environment and have complicated effects on human health, causing organ damage and even cancer (22–24). Cd has been found in at least 1,014 of the 1,669 most serious hazardous waste sites in the nation by the Environmental Protection Agency of the United States. The number of sites with Cd may increase as more waste disposal sites are evaluated (25). Frank et al. showed that Pb exposure was a widespread public health problem in the United States and this contamination affected all aspects of people’s daily lives, including electronics, cosmetics, etc. (26). Co is more abundant on the Earth’s surface than Pb and Cd (27). As a key component of electric vehicle batteries, Co has become more prevalent in people’s lives with the development of new power trams. The concentration of Co rises with an increase in municipal solid waste (28). Cd, Co, and Pb can induce an inflammatory response in the body through oxidative stress, disrupting the normal functioning of the immune system (29–32). The experimental result has demonstrated that rats exposed to Pb develop neuroinflammation of microglia in their brains, leading to adverse neurodevelopmental outcomes (33). Lin et al. have demonstrated that Cd exposure induced an inflammatory response in the mouse heart by disrupting the secretion of inflammatory cytokines through the NF-κB signaling pathway (34). Cd was also able to promote inflammatory processes by making macrophages more susceptible to inflammatory polarization (35). Co induced a HIF-1α-dependent metabolic shift from oxidative phosphorylation to glycolysis in macrophages, which played an important role in activating inflammatory responses (36).

Given the recent interest in SII as a marker of inflammation, the importance of investigating the influences cannot be overstated. Considering that metals and PAH pollutants are commonly distributed in life, it is crucial to explore the effects of metals and PAHs on SII. Therefore, we applied data from the National Health and Nutrition Examination Survey (NHANES) to explore the effects of several common pollutants such as Cd, Co, Pb, and PAHs on SII, and to analyze the effects of mixed exposures in addition to understanding the role of single pollutants.

## 2 Materials and methods

### 2.1 Study population

**Figure 1:**
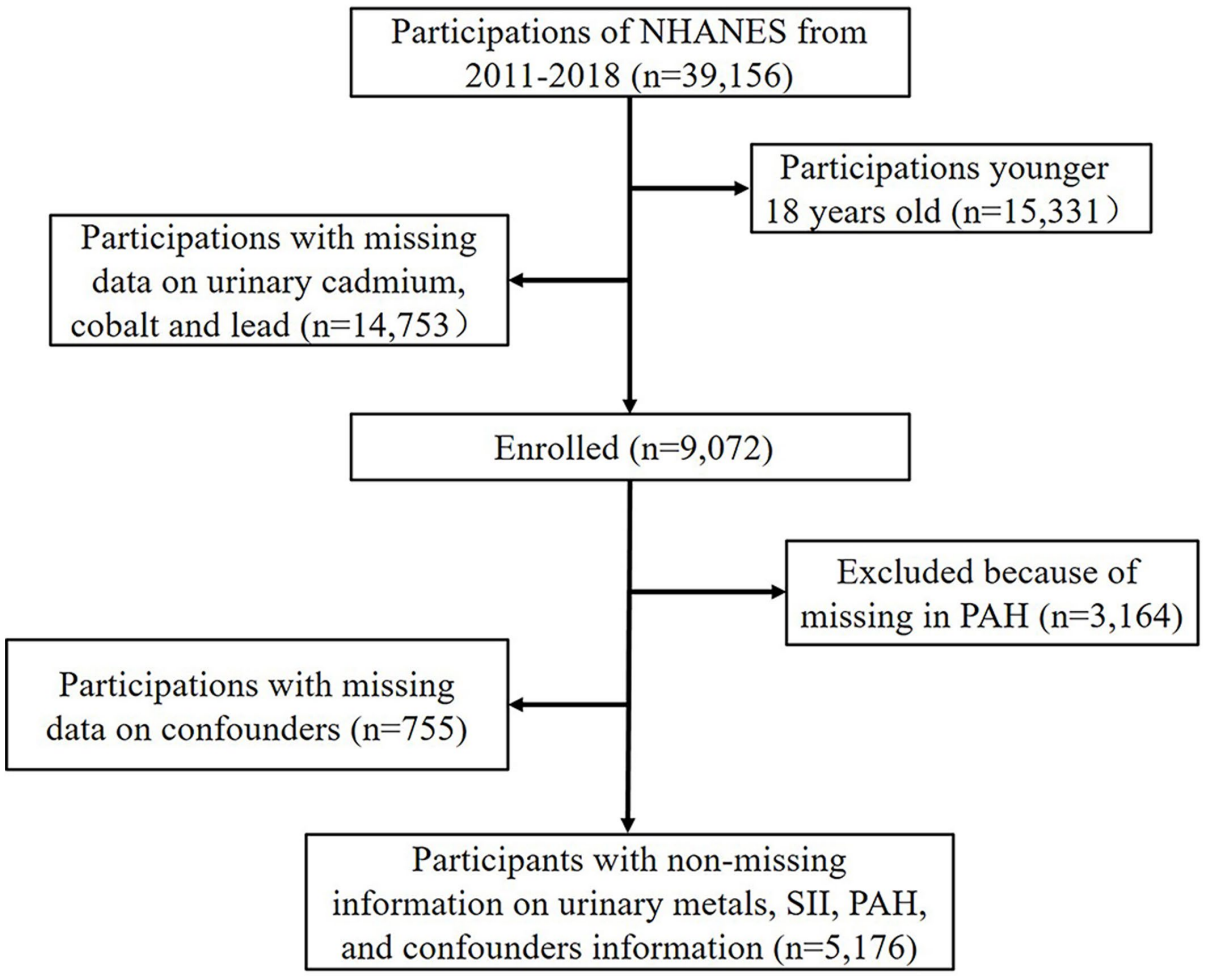
Schematic diagram of study methodology in NHANES 2011–2018.

In this study, we used data from NHAENS, a population-based survey that investigated the health and nutritional status of different ethnic groups in the United States (37). The survey was approved by the National Center for Health Statistics Research Ethics Review Board and conducted in accordance with local legislation and institutional requirements. All participants provided their written informed consent to participate in this study. Through a complex multi-stage design, the study collected basic population information, biological samples, and examination data from a representative population (38). Information about the NHANES study design, data collection process, data weighting, and participants’ informed consent is obtainable in the National Center for Health Statistics public database (39). Our analysis combined data from the 2011–2012, 2013–2014, 2015–2016, and 2017–2018 NHANES cycles. A total of 39,156 subjects were initially enrolled, with participants younger than 18 years old (*n* = 15,331), missing information on urinary Cd, Co, and Pb (*n* = 14,753), missing data on PAHs (*n* = 3,164), and missing information on confounders (*n* = 755) excluded from the study. Finally, 5,176 appropriate participants were included in the study. The participant selection flowchart was shown in Figure 1.

### 2.2 Contaminants assessment

According to the standards of the Laboratory Sciences Division, National Center for Environmental Health, Atlanta, Georgia, urinary Cd, Co, and Pb were analyzed by inductively coupled plasma dynamic reaction cell mass spectrometry. Metal concentrations below the limit of detection (LOD) were calculated by dividing the LOD by the root sign 2 (40). Urinary monohydroxylated metabolites of PAH were commonly studied by researchers as stable biomarkers of PAH exposure. Although different techniques were used in different NHANES cycles and a slight error existed between techniques, 1-hydroxynaphthalene (1-OHN), 2-hydroxynaphthalene (2-OHN), 3-hydroxyfluorene (3-OHF), 2-hydroxyfluorene (2-OHF), 1-hydroxyphenanthrene (1-OHPhe), and 1-hydroxypyrene (1-OHPyr) were measured in all four cycles from 2011 to 2018, and ultimately, urinary Cd, Co, Pb and six PAHs were included in the current study.

### 2.3 Outcomes

Blood samples were transported to the NHANES Mobile Examination Centers for final determination. Detailed descriptions of the sample collection, transportation, and processing were presented in the NHANES Laboratory/Medical Technician Procedures Manual. Automated blood analysis equipment was used to measure lymphocyte, neutrophil, and platelet counts, and finally, the SII level was calculated by multiplying the platelet count by the neutrophil count/lymphocyte count (5).

### 2.4 Other covariates

The other covariates included age, gender, body mass index (BMI), race, marriage, education level, drinking, smoking, physical activity, and sedentary. According to the NHANES statement, information on basic demographic characteristics of the study population and lifestyle information was collected by professionals. Age was categorized into <40 years, 40–60 years, and > 60 years. BMI was categorized into normal (18.5–24.9 kg/m^2^) and abnormal (< 18.5 kg/m^2^ or ≥ 25.0 kg/m^2^). Marriage was categorized into married, unmarried, and other status. Since participants younger than 20 were not asked for marital information on the survey, we uniformly categorized the marital status of those younger than 20 as unmarried. Race was categorized into Hispanic, non-Hispanic white, non-Hispanic black, and non-Hispanic other. Education level was categorized into less than high school education, high school education, and more than high school education. Alcohol consumption was categorized into never drinking, ever drinking, and current drinking, and smoking was categorized as never smoking, ever smoking, and current smoking (41). Physical activity was categorized as <100 min MVPA(moderate to vigorous physical activity), and ≥ 100 min MVPA. 75 min of vigorous physical activity was equal to 150 min of moderate physical activity (42). Physical activity time was recorded as the time during the week that the participant engaged in any vigorous or moderate activity that would result in changes in breathing or heart rate. Sedentary was categorized as <360 min, and ≥ 360 min. Sedentary time was defined as all the time the participant spent sitting during the day, including time spent sitting working, learning, and resting. Due to the complex sampling methods of the NHANES database, the NHANES cycle was included as a covariate to weigh the different years of sampling.

### 2.5 Statistical analysis

SPSS 24.0 and R 4.2.0 were used for statistical description and analysis. All data were expressed as percentages for categorical variables and P50 (P25, P75) for skewed variables. The chi-square and non-parametric tests were used to compare the differences in fundamental information. The correlation test was used to analyze the relationship between the log-transformed concentration of Cd, Co, Pb, and PAHs. The single contaminant effect on SII used the linear regression model and restricted cubic spline (RCS) to analyze, with correcting for confounding factors such as age, gender, race, marriage, education, smoking, drinking, physical activity, and sedentary. To explore the multiple contaminants effect on SII, a Bayesian Kernel Machine Regression (BKMR) analysis was performed. The modeling of BKMR consists of three parts: the regression analysis layer, the kernel function layer, and variable selection (43). It does not need to set up parametric expressions, allows for nonlinear effects and interactions, generates a kernel function based on the exposure variables put into the model, and then generates the relationship curves (dose–response curves) between the mixture components and the exposure variables by using the Bayesian sampling and analytical methods. The formula of BKMR is as follows:


$$Y_{i}=h\left(z_{i1},\ldots .,z_{iM}\right)+x_{i}\beta +\epsilon_{i}$$

*Y*: the event for individual i; *z*: the exposure factor; χ: the covariate; *h*(): exposure response function; *β*: effect on covariates; *ϵ*: residual term. We also used generalized weighted quantile sum (gWQS) regression analysis to explore the effect weight of Cd, Co, Pb, and six PAHs on SII. The gWQS is used for multivariate regression on common high-dimensional datasets such as environmental exposures, genomics, and metabolomics studies. The model builds a weighted index that estimates the mixed effect of all predictor variables on the outcome, which can then be used in a regression model with associated covariates to test the association of the index with the dependent variable or outcome (44). The contribution of each individual predictor to the whole can then be assessed by the weighting index assigned to each variable by the model. Finally, to assess the different impacts of Cd, Co, Pb, and six PAHs on SII in males and females, the interaction analysis was used. Cd, Co, Pb, and six PAHs were divided into high and low concentration groups by median, and then the interaction between pollutants and males and females was analyzed to determine whether there was a statistically significant effect of pollutants on SII. The distribution of males and females in both the high and low concentration groups was counted in the results of the interaction. In this study, we used R language packages such as “compareGroups,” “rms,” “QualInt,” etc. Two-sided test with a test level of *α* = 0.05 was used.

## 3 Results

### 3.1 Study population characteristics

The general characteristics of the study participants were presented in Table 1. The percentage of males and females in this study population was 51.3 and 48.7%, respectively. 76.1% of the participants were Non-Hispanic. Over 70% of the participants reported having an abnormal BMI. 76.7% of the participants had a high school degree or higher, and this percentage was higher for females than for males. The percentage of current smokers was 31.4% for males and 21.7% for females. The percentage of current drinkers was 73.3% for males and 62.9% for females. There was no statistically significant difference between the time of physical activity and sedentary in males and females. All six PAHs were present in higher concentrations in the urine of males than females. The level of Co in urine is higher in females than in males (0.37 μg/L vs. 0.36 μg/L), and the distribution of Pb was opposite in males and females (0.42 μg/L vs. 0.31 μg/L). A difference existed in the distribution of SII by gender, with males being higher than females (467 10^3^/μL vs. 430 10^3^/μL). The correlation analysis between the concentrations of the three metals and the six PAHs after log transformation was shown in Supplementary Figure 1.

**Table 1:** Characteristics of study population in NHANES 2011–2018 (*n* = 5,176).

	Total (*n* = 5,176)	Male (*n* = 2,656)	Female (*n* = 2,520)	*p*
Age (%)				0.434
<40 years	1947 (37.6)	1,020 (38.4)	927 (36.8)	
40–60 years	1760 (34.0)	885 (33.3)	875 (34.7)	
>60 years	1,469 (28.4)	751 (28.3)	718 (28.5)	
Race (%)				0.027
Mexican American	689 (13.3)	332 (12.5)	357 (14.2)	
Other Hispanic	553 (10.7)	260 (9.8)	293 (11.6)	
Non-Hispanic White	2042 (39.4)	1,049 (39.5)	993 (39.4)	
Non-Hispanic Black	1,147 (22.2)	618 (23.3)	529 (21.0)	
Non-Hispanic other	745 (14.4)	397 (14.9)	348 (13.8)	
BMI (%)				0.781
Normal	1,518 (29.3)	784 (29.5)	734 (29.1)	
Abnormal	3,658 (70.7)	1872 (70.5)	1786 (70.9)	
Marriage (%)				<0.001
Married	2,442 (47.2)	1,379 (51.9)	1,063 (42.2)	
Unmarried	1,211 (23.4)	639 (24.1)	572 (22.7)	
Other status	1,523 (29.4)	638 (24.0)	885 (35.1)	
Education (%)				<0.001
Less than high school degree	1,203 (23.2)	660 (24.8)	543 (21.6)	
High school degree	1,187 (23.0)	637 (24.0)	550 (21.8)	
More than high school degree	2,786 (53.8)	1,359 (51.2)	1,427 (56.6)	
Smoking (%)				<0.001
Current	1,383 (26.7)	835 (31.4)	548 (21.8)	
Ever	1,088 (21.0)	677 (25.5)	411 (16.3)	
Never	2,705 (52.3)	1,144 (43.1)	1,561 (61.9)	
Drinking (%)				<0.001
Current	3,532 (68.2)	1948 (73.3)	1,584 (62.9)	
Ever	845 (16.3)	465 (17.5)	380 (15.1)	
Never	799 (15.5)	243 (9.2)	556 (22.0)	
Physical activity (%)				0.071
<100 min MVPA	2,909 (56.2)	1,460 (55.0)	1,449 (57.5)	
≥100 min MVPA	2,267 (43.8)	1,196 (45.0)	1,071 (42.5)	
Sedentary (%)				0.364
<360 min	2,304 (44.5)	1,199 (45.1)	1,105 (43.8)	
≥360 min	2,872 (55.5)	1,457 (54.9)	1,415 (56.2)	
Cd (μg/L)	0.20 (0.10, 0.43)	0.20 (0.10, 0.42)	0.21 (0.09, 0.44)	0.924
Co (μg/L)	0.36 (0.21, 0.58)	0.36 (0.21, 0.55)	0.37 (0.21, 0.65)	<0.001
Pb (μg/L)	0.36 (0.20, 0.65)	0.42 (0.23, 0.74)	0.31 (0.17, 0.55)	<0.001
1-OHN (ng/L)	1720 (681, 6,725)	2,129 (838, 7,801)	1,388 (572, 5,510)	<0.001
2-OHN (ng/L)	5,776 (2,452, 12,995)	5,910 (2,650, 13,295)	5,608 (2,249, 12,604)	0.008
3-OHF (ng/L)	90 (40, 330)	117 (50, 432)	69 (31, 216)	<0.001
2-OHF (ng/L)	227 (104, 648)	272 (129, 808)	178 (84, 500)	<0.001
1-OHPhe (ng/L)	114 (61, 217)	124 (66, 236)	107 (56, 199)	<0.001
1-OHPyr (ng/L)	119 (50, 238)	127 (65, 258)	111 (50, 222)	<0.001
Platelet count (10^3^/μL)	232 (197, 271)	221 (188, 257)	244 (208, 288)	<0.001
Neutrophils count (10^3^/μL)	4.00 (3.10, 5.20)	3.90 (3.10, 5.00)	4.10 (3.10, 5.30)	0.001
Lymphocyte count (10^3^/μL)	2.10 (1.70, 2.60)	2.00 (1.60, 2.50)	2.20 (1.70, 2.70)	<0.001
SII (10^3^/μL)	450 (316, 636)	430 (305, 614)	467 (330, 662)	<0.001

BMI, body mass index; MVPA, moderate to vigorous physical activity; Cd, cadmium; Co, cobalt; Pb, lead; 1-OHN, 1-hydroxynaphthalene; 2-OHN, 2-hydroxynaphthalene; 3-OHF, 3-hydroxyfluorene; 2-OHF, 2-hydroxyfluorene; 1-OHPhe, 1-hydroxyphenanthrene; 1-OHPyr, 1-hydroxypyrene; SII, Systemic Immunity Inflammation Index.

### 3.2 Single contaminant and SII

**Figure 2:**
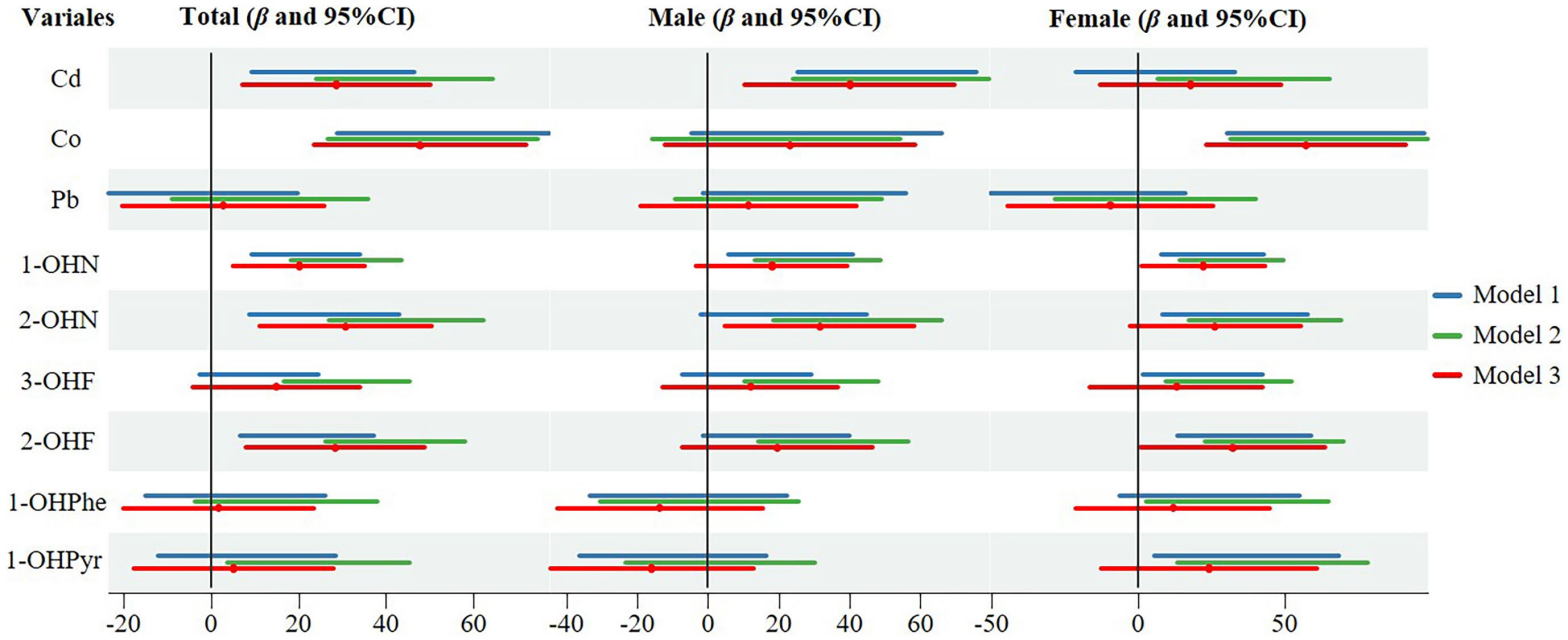
Linear regression results of log-transformed concentration of individual contaminants with SII. Model 1: no covariates were adjusted; Model 2: age, gender, and race were adjusted (only age and race in male or female); Model 3: age, gender, race, BMI, marriage, education, smoking, drinking, physical activity, sedentary, and NHANES cycles were adjusted.

Linear regression results of log-transformed concentration of contaminants with SII were shown in Figure 2. In model 3, after adjusting for age, gender, race, BMI, marriage, education, smoking, drinking, physical activity, sedentary, and NHANES cycles, Cd, Co, 1-OHN, 2-OHN and 2-OHF were positive with SII in the total population, with the coefficients of *β* being 28.413 (95%CI: 6.985, 49.841), 47.675 (95%CI: 23.372, 71.979), 19.911 (95%CI: 4.984, 34.837), 30.416 (95%CI: 10.741, 50.090) and 28.041 (95%CI: 7.628, 48.454), respectively. In males, after adjusting for all covariates, only Cd and 2-OHN were positive with SII. In females, after adjusting for all covariates, Co, 1-OHN, and 2-OHF were positive with SII and the *β* coefficient were 57.496 (95%CI: 23.393, 91.599), 21.823 (95%CI: 0.784, 42.862) and 31.915 (95%CI: 0.529, 63.301), respectively. RCS analyses showed no significant nonlinear associations between all contaminants and SII (with all *p* for the nonlinearity of >0.05), while the associations between Cd, Co, and SII were statistically significant (with *p* for overall of 0.025 and < 0.001; Supplementary Figure 2).

### 3.3 Multiple contaminant and SII

**Figure 3:**
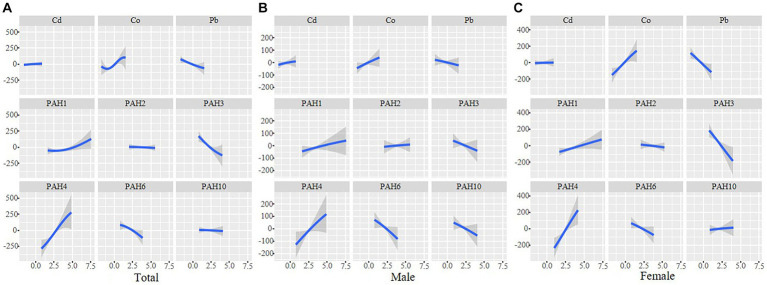
Univariate exposure–response functions and 95% confidence interval for association between single metal exposure when other metals exposure are fixed at the median in total population **(A)**, males **(B)** and females **(C)**. Models were adjusted for age, gender, race, BMI, marriage, education, smoking, drinking, physical activity, sedentary, and NHANES cycles.

**Figure 4:**
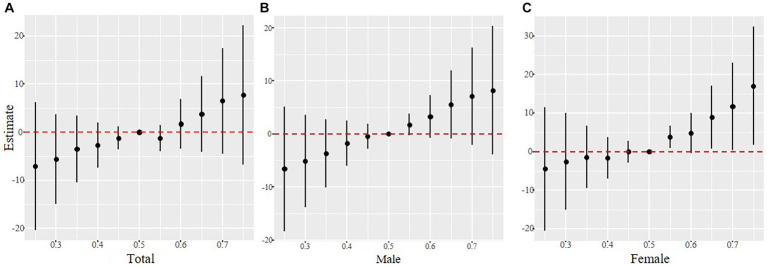
The joint effects of contaminant mixtures on SII were estimated in total population **(A)**, males **(B)** and females **(C)**, when all the contaminants at particular percentiles were compared to all the metals at their 50th percentile. Models were adjusted for age, gender, race, BMI, marriage, education, smoking, drinking, physical activity, sedentary, and NHANES cycles.

**Figure 5:**
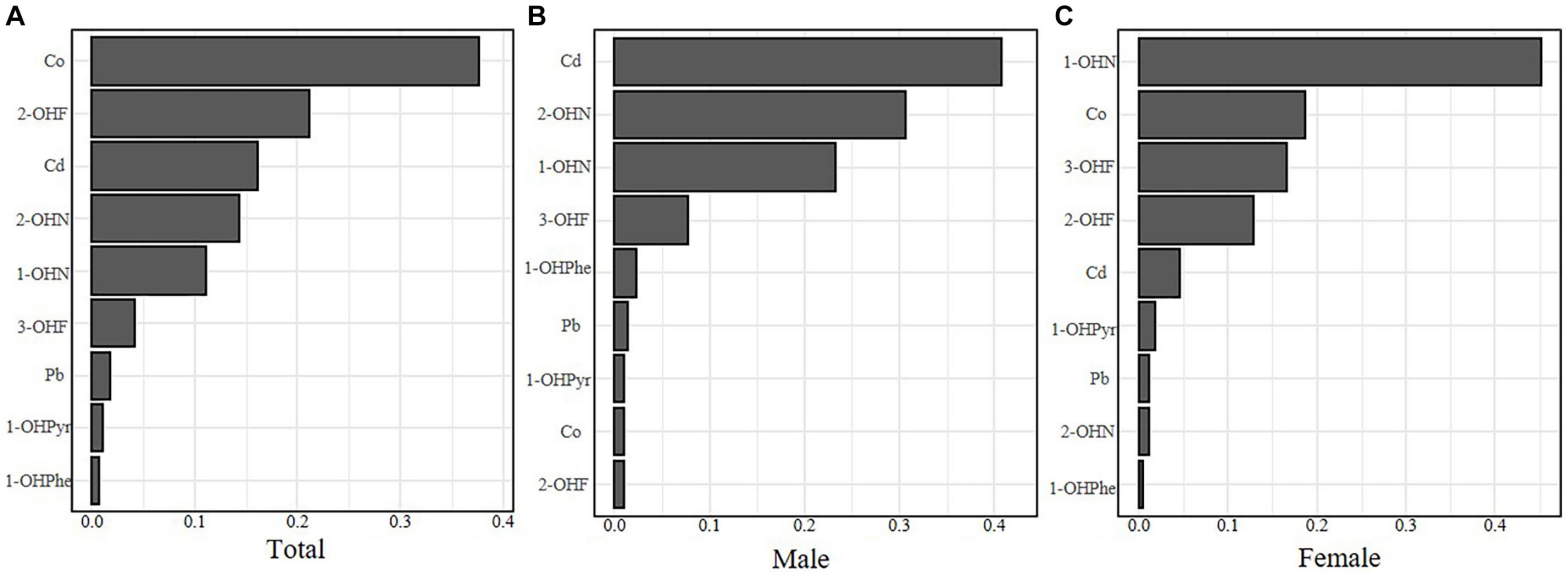
The effect weight of contaminant mixtures on SII of the total population **(A)**, males **(B)** and females **(C)**. Models were adjusted for age, gender, race, BMI, marriage, education, smoking, drinking, physical activity, sedentary, and NHANES cycles.

As shown in Figure 3, Co, 1-OHN, and 2-OHF were positively correlated with SII when other contaminants were fixed at the median, and Pb, 3-OHF, and 1-OHPhe had a negative association with SII in the total participants. The joint effect of Cd, Co, Pb, and six PAHs on SII was presented in Figure 4. Compared with the 50th percentile, the level of SII tended to increase with the increase of contaminant concentration, and the overall effects of the total population and males were not significant, but a significance existed in the overall effect of females when contaminant levels exceeded 50%. As shown in Supplementary Figure 3, to examine the contribution of a single pollutant to the overall effect with mixed exposures, we fixed the other pollutants at the 25th, 50th, or 75th percentile and determined significant associations between Co, Pb, 3-OHF, and 2-OHF and SII in the total population and females. Our findings suggest that increased exposure to 2-OHF is associated with higher levels of SII, whereas 3-OHF is associated with lower levels of SII. Results of the effect weight of contaminant mixtures on SII derived from the gWQS model were presented in Figure 5. In the total population, the top three contaminants with the highest effect weights on SII were Co [weight (w) = 0.385], 2-OHF (w = 0.214), and Cd (w = 0.130). In males, the top three contaminants were Cd (w = 0.429), 2-OHN (w = 0.342), and 1-OHN (w = 0.229). In females, the three contaminants were 1-OHN (w = 0.457), Co (w = 0.218), and 3-OHF (w = 0.129). The impact of mixed pollutant exposure on SII in the gWQS results was presented in Supplementary Table 1.

### 3.4 Interaction analysis

Finally, the interaction analyses were conducted to explore the interaction between gender and contaminants on SII. As shown in Supplementary Figure 4, the interaction effect between the concentrations of Cd and gender on SII was statistically significant (*p* for interaction = 0.023), and in combination with the results of Figure 5, the conclusion was that the low concentrations of Cd had an elevation effect on SII in males. The interaction effect between the concentrations of Co and gender on SII was almost statistically significant (*p* for interaction = 0.057), and the high concentration of Co had an elevation effect on SII in females.

## 4 Discussion

SII is negatively correlated with the recovery of many diseases. Wang et al. conducted a retrospective study to investigate the effect of SII on the prognosis of patients with advanced lung cancer and found that SlI (HR = 1.960) was an independent factor affecting the progression-free survival of patients (45). Another prospective cohort study showed that SII > 663 10^3^/μL in the baseline was associated with an increased risk of cervical cancer recurrence and death (46). Jiang et al. investigated the association of arthritic disease with new inflammatory markers and found that SII was significantly higher in the acute ventilatory arthritis group than in the healthy population group (*p* < 0.05) (47). A study based on clinical trial data exposes that patients with cardiovascular disease in the high SII group had significantly lower survival rates than those in the low SII group (48). Considering the importance of novel biomarkers in disease research, the comprehensive discovery of factors associated with SII is of great clinical significance.

By reviewing the literature, previous epidemiologic studies have primarily emphasized the relationship between individual categories of pollutants and SII, and the complications of mixed exposures to different pollutants have not been elucidated. An epidemiologic study conducted in a population in northwest China found that after combined exposure to Cd and Pb, subjects in the high co-exposure group had higher levels of SII compared with those in the medium and low co-exposure groups, suggesting that co-exposure to Cd and Pb is associated with SII and that the immune-inflammatory response is exacerbated with rising co-exposure to Cd and Pb (49). Based on 2015–2016 NHANES data, Zhong et al. used generalized linear models to assess the independent and joint effects of SII with blood Mn, Cd, and Pb levels, respectively (50). More recently, using NHANES data, sun et al. similarly used univariate models to evaluate the linear and nonlinear relationships between single metal exposures and SII, and analyzed the combined effects of mixed metal exposures on SII (11). In addition, a study investigating the relationship between co-exposure to PAH and phthalates and SII in children aged 4–12 years showed that concurrent exposure to PAH and phthalates was related to increased inflammation in children, with 1-OHPyr having an important role (51). SII, a novel marker of systemic inflammation, has been demonstrated to be strongly associated with the development of various diseases. Given the widespread co-existence of multiple pollutants in the environment, it is of great significance for this study to investigate the combined exposure with Cd, Co, Pb, and PAHs.

In this study, we examined the relationship between Cd, Co, Pb, 1-OHN, 2-OHN, 3-OHF, 2-OHF, 1-OHPhe, 1-OHPyr, and SII among U.S. adults, exploring the differential effects of these pollutants in men and women. In the analysis between a single contaminant and SII, we found that Cd, Co, 1-OHN, 2-OHN, and 2-OHF were positive with SII in the total population. According to previous reports, the results of this study are reasonable and evidence-based. Chronic exposure to Cd resulted in a significant increase in platelet counts and a decrease in lymphocyte counts in mice (52). Akbar et al. showed that Co was not directly cytotoxic to lymphocytes but might affect intracellular molecular homeostasis and thus inhibit lymphocyte proliferation (53). Exposure to diesel exhaust containing high levels of PAHs triggers an increase in neutrophils and lymphocytes (54). Whereas, there have also been experiments with different conclusions. To explore chronic exposure to PAHs and changes in platelet indices, Cui et al. used longitudinal data from repeated-measures analyses and found a linearly correlated dose–response relationship between 1-OHN and platelets (55). The results of a cross-sectional study revealed that neutrophil and monocyte counts were lower in the high-concentration Cd group compared to the low-concentration Cd group (56). More studies need to be conducted to explore the association between metals and PAHs exposure and SII. Interestingly, in males, only Cd and 2-OHN had a positive association with SII, while in females, these were Co, 1-OHN, and 2-OHF, which might be because different pollutants exert different effects in males and females. Yang et al. found that after exposure to Cd, female mice gained normal body weight while male mice lost significant body weight, which was related to the different responses of gut microbiota and adrenocorticotropic hormone homeostasis in female and male mice (57). Li et al. showed that lipid metabolites in mice exhibited different susceptibility and trends in male and female mice after exposure to PAHs (58). Ferrari et al. used Gaussian to identify effects and interactions between pollutants and found negative effects of Co exposure to be stronger for males than females (59). The specific mechanisms by which pollutants play different effects on males and females remain to be explored in further animal or cellular experiments.

When exploring the association between mixed exposure to Cd, Co, Pb, six PAHs, and SII, the results of BKMR showed that Pb, 3-OHF, and 1-OHPhe had a negative association with SII in the total, male and female populations, which was different from the result of a single pollutant. We hypothesize that this may be due to the interaction between multiple contaminants, leading to different conclusions than single contaminants. The joint effect of contaminant mixtures on SII was positive when contaminant levels exceeded 50% and this was only statistically significant in females. According to the interaction analysis results, the interaction between the low Cd concentration group, the high Co concentration group, and gender was statistically significant for SII, which meant that the two groups did not play the same role in males or females. The result of gWQS model showed that in the total population, Co had the main role on SII, whereas in males and females, those were Cd and 1-OHN, respectively. Combining the above conclusions, we concluded that low concentrations of Cd had a greater elevation effect on SII in males than in females, and the high concentration of Co had a stronger elevation effect on SII in females than in males. Not sufficient scientific evidence existed to demonstrate that Cd and Co had significantly different effects on the immune systems in males and females. However, sensitivity to Cd and Co was different in males and females (60–62), suggesting that changes in SII in males and females at different concentrations of Cd and Co were likely to be inconsistent. Lamtai et al. showed that in male rats, all doses of Cd triggered depressive-like behavior, whereas in female rats, only those receiving high doses exhibited depressive-like behavior (63). Isaksson et al. found a higher percentage of positive skin reactions to Co in females than in males (64). The above results indicated that when we study the association between metal exposure and inflammatory markers, we should not only focus on how different pollutants affect males and females differently but also study the effects at different concentrations.

Several innovative points are addressed in this study. First, we explored for the first time the association between mixed exposure to metals and PAHs and SII, elucidating the different roles played by different pollutants in males and females. Second, we utilized models such as BKMR and gWQS, which are specifically designed to analyze the association between mixed exposures and outcomes, making our results accurate and reliable. Finally, the study population included in this survey was more than 5,000 people, which is a relatively sufficient sample size to stabilize the results of this study. This study also has some limitations. First, the ability of this study to verify causality is weaker than cohort studies. Therefore the generalizability of this article remains to be confirmed by more cohort studies. Second, this study only explored the association of mixed exposure with SII, which could be studied in combination with more inflammatory markers.

## 5 Conclusion

Based on the results of the present study, we found a positive association of mixed exposure to Cd, Co, Pb, and six PAHs with the novel inflammatory marker SII, which occurred mainly in females. In the total population, males, and females, the pollutants with the greatest effect on SII were Co, Cd, and 1-OHN, respectively.

## Data availability statement

The original contributions presented in the study are included in the article/Supplementary material, further inquiries can be directed to the corresponding author/s.

## Ethics statement

The studies involving humans were approved by the National Center for Health Statistics Research Ethics Review Board. The studies were conducted in accordance with the local legislation and institutional requirements. The participants provided their written informed consent to participate in this study.

## Author contributions

JN: Writing – review & editing. DL: Writing – review & editing. ZH: Methodology, Writing – original draft. CX: Visualization, Writing – review & editing. MH: Visualization, Writing – review & editing. WZ: Conceptualization, Data curation, Software, Writing – original draft.
