# Supplementary material for: The single and mixed impacts of cadmium, cobalt, lead, and PAHs on systemic immunity inflammation index in male and female
Source: Front Public Health. 2024 Feb 15;12:1356459. doi: 10.3389/fpubh.2024.1356459 (PMC10902425; doi:10.3389/fpubh.2024.1356459)
Supplement: Supplementary file 1 [file Data_Sheet_1.docx]

Supplementary Material

# Supplementary Data

The datasets generated and/or analysed during the current study are available in the NHANES repository, https://www.cdc.gov/nchs/nhanes/index.htm.

# Supplementary Figures and Tables

## Supplementary Figures


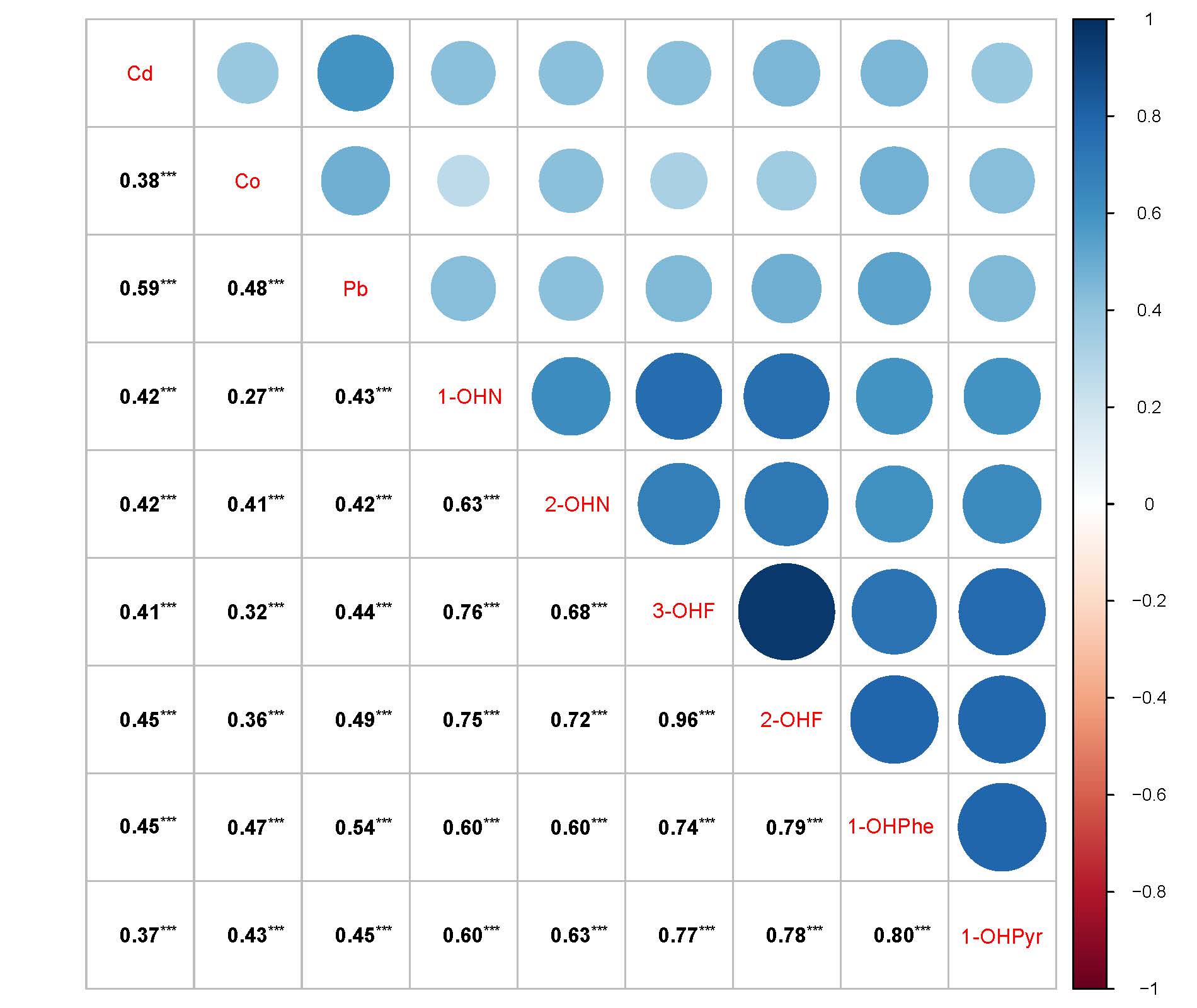


**Supplementary Figure 1.** The correlation analysis between the log-transformed concentrations of Cd, Co, Pb, and six PAHs.


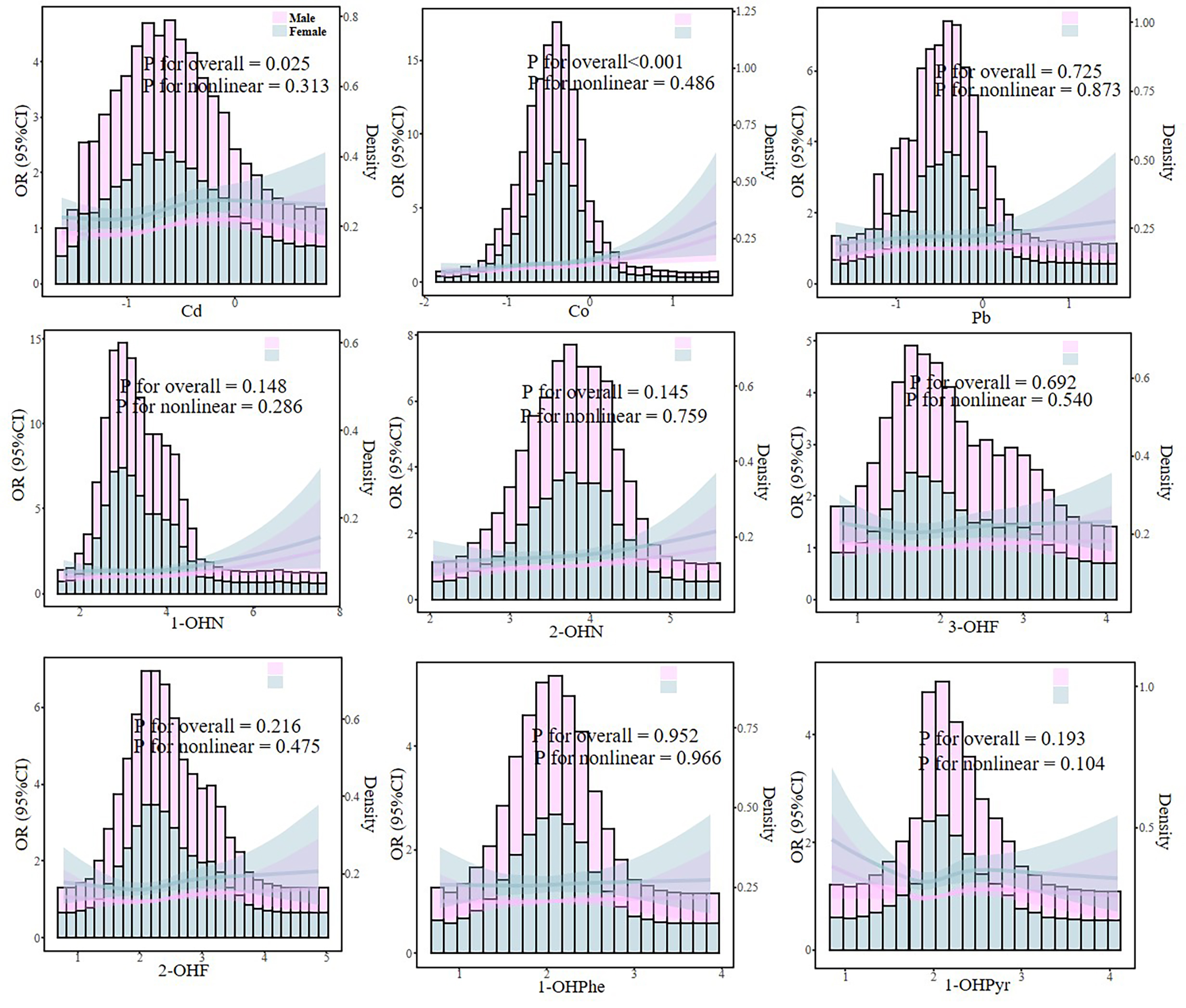


**Supplementary Figure 2.** Adjusted restricted cubic spline (RCS) for the association between contaminants and SII. Models were adjusted for age, gender, race, BMI, marriage, education, smoking, drinking, physical activity, sedentary, and NHANES cycles.


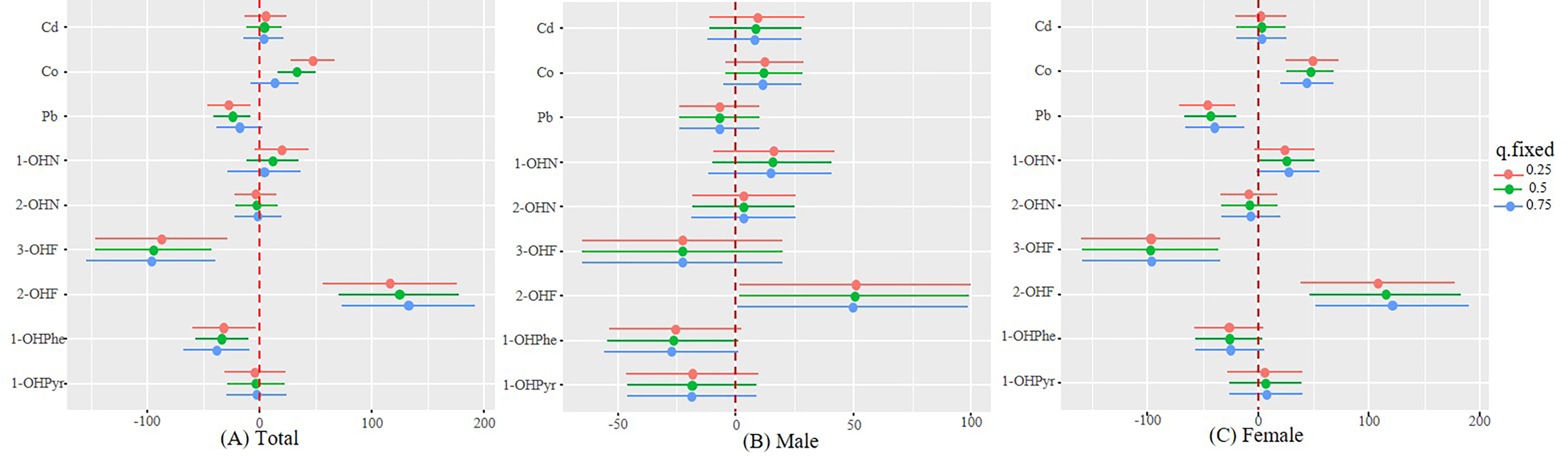


**Supplementary Figure 3.** The 75th percentile of the individual pollutant was compared to its 25th percentile in total population (A), males (B) and females (C), with the other essential pollutants at their 25th, 50th, or 75th percentile. Models were adjusted for age, gender, race, BMI, marriage, education, smoking, drinking, physical activity, sedentary, and NHANES cycles.


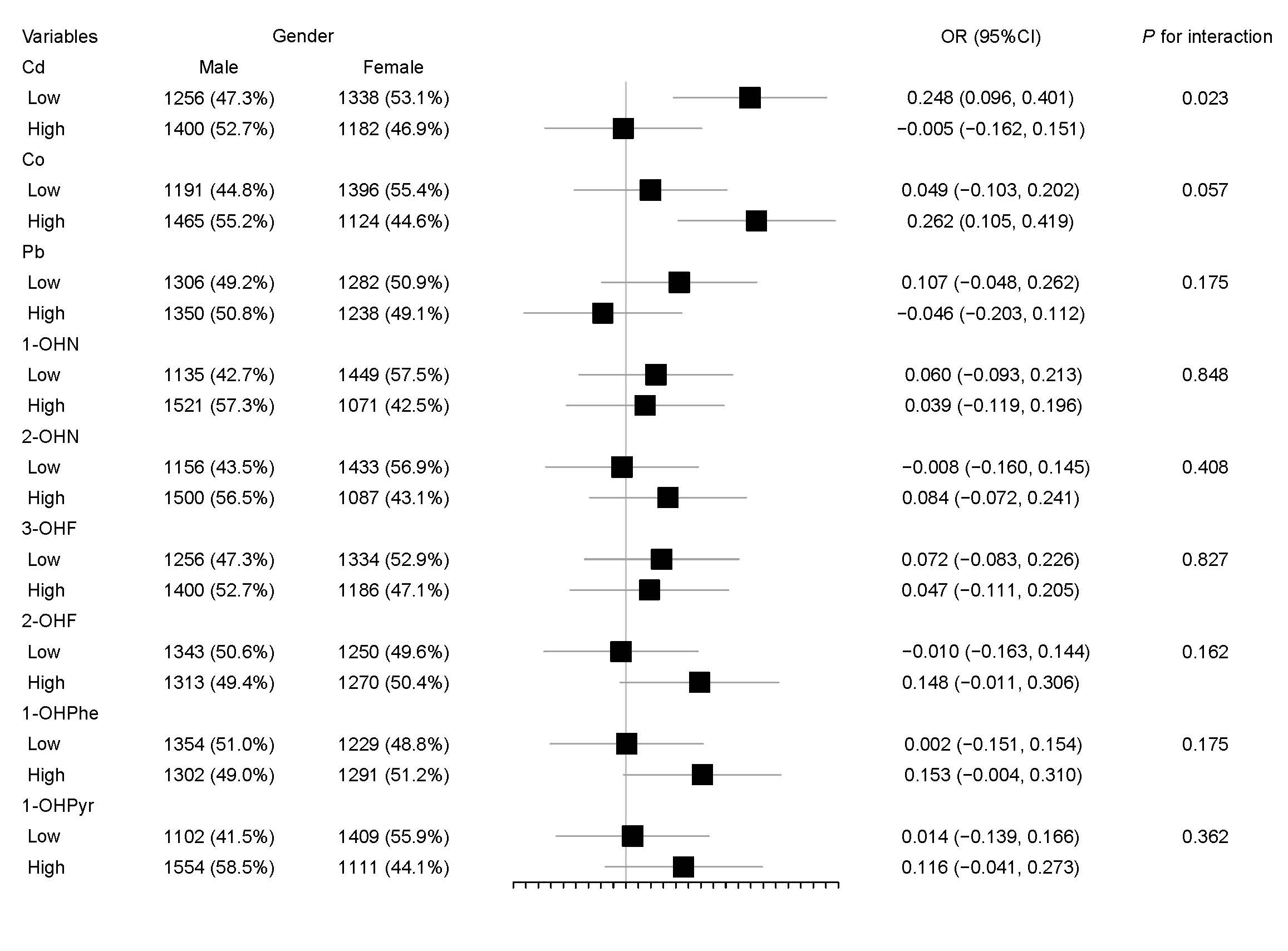


**Supplementary Figure 4.** The interaction effects between contaminants and gender on SII.

## Supplementary Tables

**Supplementary Table 1** The results of the gWQS between exposure to Cd, Co, Pb, six PAHs and the level of SII.

| Groups | Estimate | Std. Error | t value | *P* |
| --- | --- | --- | --- | --- |
| Total | 6.460 | 2.934 | 2.201 | 0.028 |
| Male | 4.109 | 4.037 | 1.018 | 0.309 |
| Female | 10.169 | 4.106 | 2.477 | 0.013 |

Models were adjusted for age, gender, race, BMI, marriage, education, smoking, drinking, physical activity, sedentary, and NHANES cycles.
